# Pathophysiology of trauma-induced coagulopathy: disseminated intravascular coagulation with the fibrinolytic phenotype

**DOI:** 10.1186/s40560-016-0200-1

**Published:** 2017-01-31

**Authors:** Mineji Hayakawa

**Affiliations:** Emergency and Critical Care Center, Hokkaido University Hospital, N14W5 Kita-ku, Sapporo, 060-8648 Japan

**Keywords:** Coagulopathy, Disseminated intravascular coagulation, Fibrinolysis, Massive bleeding, Transfusion, Fibrinogen, Trauma

## Abstract

In severe trauma patients, coagulopathy is frequently observed in the acute phase of trauma. Trauma-induced coagulopathy is coagulopathy caused by the trauma itself. The pathophysiology of trauma-induced coagulopathy consists of coagulation activation, hyperfibrino(geno)lysis, and consumption coagulopathy. These pathophysiological mechanisms are the characteristics to DIC with the fibrinolytic phenotype.

## Background

**Fig. 1:**
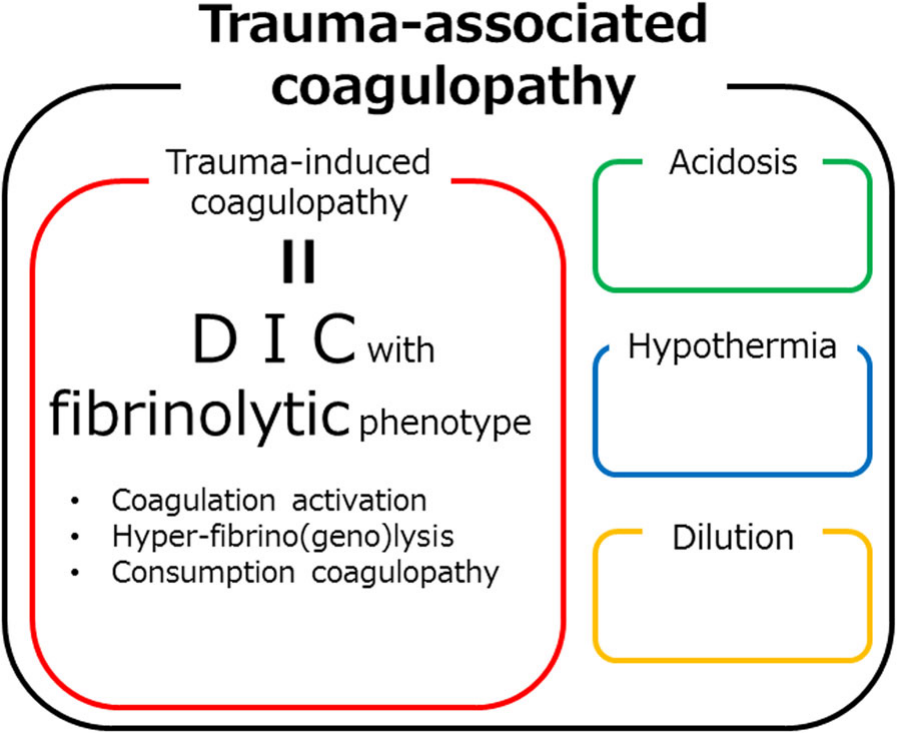
Trauma-associated coagulopathy and trauma-induced coagulopathy. Trauma-associated coagulopathy is caused by multiple factors and includes trauma-induced coagulopathy, which is caused by trauma itself.

In severe trauma patients, coagulopathy is frequently observed in the acute phase of trauma, with profound effects on outcome [1–7]. This coagulopathy is caused by multiple factors associated with the trauma itself as well as certain interventions [8–12] and has been described with various terms. In this manuscript, we refer to the coagulopathy caused by diverse trauma-associated factors as “trauma-associated coagulopathy” and the coagulopathy caused by the trauma itself as “trauma-induced coagulopathy” (Fig. 1).

### Inconsistencies in the acute coagulopathy of trauma shock theory

#### Coagulation suppression by activated protein C?

In the acute coagulopathy of trauma shock (ACoTS) theory, trauma-shock stimulates release of soluble thrombomodulin (TM) from endothelial cells [13, 14]. Soluble TM binds to thrombin to form a thrombin-TM complex, which activates protein C [13, 14], which in turn suppresses prothrombinase complex (factor Va-factor Xa complex) activity and thrombin formation [13, 14].

However, TM is a receptor of thrombin and protein C on the endothelial cell surface and regulates the coagulation and complement system [15]. Soluble TM is formed via the limited proteolysis of TM by neutrophil elastase on the endothelial cell surface [16, 17], but it has not been confirmed that soluble TM is actively secreted by endothelial cells. However, the level of soluble TM correlates with the degree of endothelial injury [16, 17]. Furthermore, soluble TM has only 20% of activity of normal TM on the endothelial cell surface [18]. Under these circumstances, the anticoagulant property of the endothelium is impaired [16, 17]. Consequently, total anticoagulant activity of TM in vessels is impaired in the acute phase of trauma [17].

#### Hyperfibrinolysis by degradation of plasminogen activator inhibitor?

In the ACoTS theory, activated protein C decomposes plasminogen activator inhibitor (PAI) [13]. Because PAI suppresses fibrinolysis, PAI degradation accelerates fibrinolysis [13].

However, activated protein C level does not increase, because, as mentioned above, total TM activity in the vessel is impaired [17]. Furthermore, plasma PAI level does not increase immediately following trauma [19]. Chapman et al. [19] indicated that total plasma PAI in severe trauma patients with hyperfibrinolysis did not increase compared to that in healthy controls. Therefore, PAI degradation does not appear to play a significant role in the pathogenesis of hyperfibrinolysis in the acute phase of trauma [17, 19].

### DIC phenotypes

We have repeatedly advocated that trauma-induced coagulopathy is a disseminated intravascular coagulation (DIC) with the fibrinolytic phenotype [12, 20–22]. However, it has been suggested that trauma-induced coagulopathy does not imply DIC [13]. We consider that this argument [13] might have resulted from a misunderstanding about DIC phenotypes.

DIC is divided into two phenotypes, the fibrinolytic and thrombotic phenotypes (Table 1) [20, 23, 24]. In critical care settings, sepsis-induced DIC is frequently observed, which is a representative of the thrombotic phenotype [24] and characterized by suppressed fibrinolysis with micro-vessel thrombosis and ischemic organ dysfunction [25]. However, trauma-induced coagulopathy, which is considered a type of DIC with the fibrinolytic phenotype, is markedly different from DIC with the thrombotic phenotype [12, 20, 21]. Coagulation activation is observed in both phenotypes of DIC. Plasma PAI suppresses fibrinolysis in DIC with the thrombotic phenotype, whereas fibrino(geno)lysis is activated by tissue-plasminogen activator (t-PA) in DIC with the fibrinolytic phenotype [24, 25]. Therefore, although sepsis-induced DIC does not lead to massive bleeding, trauma-induced DIC (fibrinolytic phenotype) in the acute phase of trauma contributes to massive bleeding and death [1–4].

**Table 1:** Characteristics of DIC phenotypes

	Fibrinolytic phenotype	Thrombotic phenotype
Representative cause	Acute phase of trauma	Sepsis
Coagulation	Activated	Activated
Fibrinolysis	Activated	Suppressed
PAI-1	Low	High
Clinical symptom	Bleeding	Organ dysfunction

*DIC* disseminated intravascular coagulation, *PAI* plasminogen activator inhibitor

### Pathophysiology of trauma-induced coagulopathy

Trauma-induced coagulopathy is generated by the following pathophysiological mechanisms:

1. Coagulation activation
  1. *Procoagulants in the systemic circulation*
  2. *Impairment of endogenous anticoagulant activity*
  3. *Thrombin generation in the systemic circulation*
2. Hyper-fibrino(geno)lysis
  1. *Acute release of t-PA-induced hyperfibrino(geno)lysis*
  2. *Coagulation activation-induced fibrino(geno)lysis*
3. Consumption coagulopathy

### Coagulation activation

#### Procoagulants in the systemic circulation

**Fig. 2:**
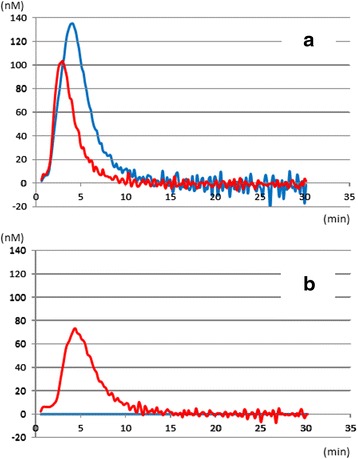
Spontaneous thrombin generation in severe trauma. **a** Stimulated thrombin generation curve. Although the amount of thrombin generation was lower in the Trauma group than the control group, time to initiation of thrombin generation and time to peak thrombin generation were shorter in the trauma group than the control group, suggesting coagulation activation. **b** Non-stimulated thrombin generation curve. Spontaneous thrombin generation was observed in the trauma group but not in the control group, demonstrating the presence of circulating procoagulants in the systemic circulation of the trauma group. *Blue line*: control group; *red line*: trauma group. (Cited as Figure 5 in our previous manuscript [27] and adapted with permission from Wolters Kluwer Health, Inc.)

In severe trauma patients, particularly those with blunt trauma, massive tissue injury accelerates thrombin generation [3, 5–7]. Previous studies showed spontaneous thrombin generation in severe trauma by using non-stimulation thrombin generation assays (Fig. 2) [26, 27]. Shortly after trauma, various procoagulants are observed in the systemic circulation, which results in this spontaneous thrombin generation (Table 2).

**Table 2:** Procoagulants circulating in the systemic circulation

Microparticles	
Platelet-derived microparticle	
Endothelial-derived microparticle	
Leukocyte-derived microparticle	
Erythrocyte-derived microparticle	
Brain-derived microparticle	
Extracellular DNA and DNA-binding proteins	
HMGB-1	
Mitochondrial DNA	
Histone-complexed DNA fragments	

The platelet-derived microparticle is a well-known procoagulant in the acute phase of trauma [28–30], and several studies have indicated that various other cell-derived microparticles are subsequently released into the systemic circulation in the acute phase of trauma, such as the leukocyte-derived [30, 31], erythrocyte-derived [31], and endothelial-derived [30, 31] microparticles. Tissue factor is exposed on the membrane of certain microparticles [30, 32, 33]. Therefore, elevation of tissue factor antigen levels in the plasma reported in previous studies [34, 35] may reflect increase of tissue factor-exposing microparticles. Recently, brain-derived microparticles were detected in brain trauma animal models [32, 33]. These brain-derived microparticles expressed neuronal or glial cell markers, procoagulant phosphatidylserine, and tissue factor [32, 33]. In addition, other injured organs may possibly release microparticles in severe trauma.

Extracellular DNA and DNA-binding proteins, which are well known as damage-associated molecular patterns, are procoagulants observed in the systemic circulation shortly after trauma [36–44]. Histone and histone-complexed DNA fragments were detected in the systemic circulation shortly after trauma and induced inflammation, coagulation activation, and organ dysfunction [36, 37]. Furthermore, early release of high mobility group box nuclear protein 1 (HMGB-1) [41–44] and mitochondrial DNA [38–40] was observed in severe trauma and induced inflammation and coagulation activation.

In penetrating trauma, especially stab wounds, there is less tissue injury than that in blunt trauma. Therefore, procoagulant production and coagulation activation are far less than that immediately following blunt trauma.

#### Impairment of endogenous anticoagulant activity

In healthy subjects, coagulation activation is regulated by endogenous anticoagulants, such as antithrombin and the TM-protein C pathway. However, in severe trauma, the endogenous anticoagulant activities are immediately impaired and dysregulation of coagulation activation is observed [1–6, 45, 46].

**Fig. 3:**
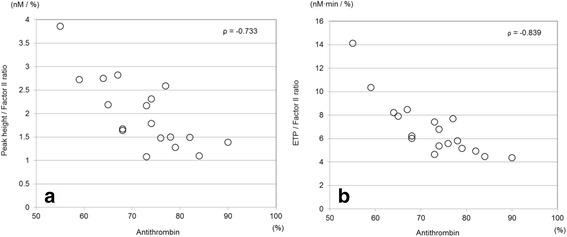
Correlations between antithrombin activity and generated thrombin. Antithrombin was significantly negatively correlated with the ratio of the peak thrombin generation level to the factor II activity (*ρ* = −0.733, *P* < 0.001). Peak height/factor II ratio, peak thrombin generation level/factor II activity. **a** Antithrombin was significantly negatively correlated with the ratio of the generated thrombin amount to the factor II activity (*ρ* = −0.839, *P* < 0.001). ETP/factor II ratio, generated thrombin amount/factor II activity. (Cited as Figure 4 in our previous manuscript [27] and adapted with permission from Wolters Kluwer Health, Inc.)

Many studies reported an early decrease in antithrombin activity in severe trauma [1–4, 45], and thrombin generation assays showed a negative correlation between antithrombin activity and generated thrombin, regardless of a decrease in prothrombin concentration (Fig. 3) [26, 27]. This result indicates that decreased antithrombin activity causes dysregulation of thrombin generation [26, 27].

Most of the TM in the body is found on vascular endothelial cells [47]. Therefore, evaluation of anticoagulation ability of TM is difficult. However, in severe trauma, endothelial damage leads to release of the TM on vascular endothelial cells as soluble TM in the systemic circulation [5, 6, 46]. Furthermore, plasma concentration of protein C decreases shortly after severe trauma [48–50]. Therefore, the anticoagulation ability of the TM-protein C pathway is impaired with resultant dysregulation of thrombin generation [2]. Although some investigators have advocated that activated protein C increases and suppresses coagulation [48–50], the increases in activated protein C (up to 10 ng/mL) did not reach sufficient concentrations to inhibit thrombin generation (70–80 ng/mL) [46, 50, 51].

#### Thrombin generation in the systemic circulation

The presence of procoagulants in the systemic circulation together with impairment of endogenous anticoagulant activities induces coagulation activation and thrombin generation [2, 27, 34, 35, 52, 53]. The half-life of thrombin is very short, which precludes measurement of plasma concentrations; therefore, other parameters have been used as evidence of thrombin generation in the systemic circulation. Soluble fibrin [2, 27] and fibrinopeptide A [34, 35, 52, 53] are considered to reflect active thrombin because these markers are formed as a result of the direct action of thrombin on fibrinogen, which is followed by fibrin formation. Early elevations of the plasma concentrations of these markers are evidence of thrombin generation in the systemic circulation and have been repeatedly reported [2, 27, 34, 35, 52, 53].

#### Hyperfibrino(geno)lysis

In severe trauma, hyperfibrino(geno)lysis, which is a combination of fibrinolysis and fibrinogenolysis, is frequently observed [1–7, 27, 34, 35, 45, 48–50, 52, 53]. This hyperfibrino(geno)lysis is caused by acute release of tissue-plasminogen activator (t-PA) and coagulation activation.

#### Shock-induced hyperfibrino(geno)lysis

One of the key enzymes in fibrino(geno)lysis is t-PA. t-PA catalyzes the cleavage of plasminogen to plasmin and thus initiates fibrin and fibrinogen degradation in plasma [54]. The main source of plasma t-PA is the Weibel-Palade body in the systemic vascular endothelial cells [54–56]. Severe shock (tissue hypoperfusion) stimulates the endothelial cells and induces release of t-PA from the Weibel-Palade bodies into the systemic circulation; this is called “acute release of t-PA” [55, 56]. Furthermore, the acute and massive t-PA release induces hyperfibrino(geno)lysis [3, 4, 12, 20, 57–62]. Thromboelastometry such as ROTEM® can detect acute release of t-PA as lysis of clots formed in test tubes [57–62].

#### Coagulation activation-induced fibrino(geno)lysis

In severe trauma, hyperfibrino(geno)lysis is frequently observed regardless of the presence of shock [3, 4, 63–69]. In particular, severe isolated head trauma, which is not usually complicated by hypotension, is a typical case in which hyperfibrino(geno)lysis may occur without shock [63, 66–69]. Hyperfibrino(geno)lysis without shock is induced by coagulation activation and is recognized by elevation of D-dimer and fibrin/fibrinogen degradation product (FDP) levels [3, 4, 63–69]. Kushimoto et al. reported [63] a correlative increase in fibrinogen degradation product and plasmin-α_2_ plasmin inhibitor complex levels. Furthermore, fibrinogen levels markedly decreased as a result of hyperfibrinogenolysis [63]. Many other studies reported that D-dimer and FDP levels increased not only in isolated head trauma [63, 66–69] but also in torso trauma regardless of the presence of shock [3, 4, 64].

In the acute phase of trauma, plasma PAI activity has not yet increased enough [19]. Therefore, although trauma-induced coagulation activation reactively causes fibrino(geno)lysis, the fibrino(geno)lysis is not suppressed by PAI [19, 65]. Furthermore, non-suppressed fibrino(geno)lysis consumes α_2_-plasmin inhibitor and the consumption of α_2_-plasmin inhibitor accelerates the dysregulation of fibrino(geno)lysis [35, 52, 63, 65, 70].

### Consumption coagulopathy

As mentioned above, in severe trauma, coagulation activation and hyperfibrino(geno)lysis are simultaneously observed. Therefore, various coagulation factors and platelets are consumed in the acute phase of trauma [1, 64, 71–75]. Consumption of coagulation factors has been repeatedly reported because this phenomenon is easy to evaluate by measurement of the coagulation factors [1, 64, 71–75]. The plasma fibrinogen level decreases more frequently and earlier than the levels of other routinely measured coagulation parameters (prothrombin time, activated partial thromboplastin time, and platelet count) [1]. Furthermore, infusion or transfusion leads more readily to dilution of fibrinogen than the other coagulation factors [10, 76]. The other coagulation factors cannot compensate for the role of fibrinogen as a unique precursor of fibrin [77, 78]; therefore, decreased fibrinogen may lead to massive bleeding and poor outcome [1, 64, 71, 72]. Other coagulation factor activities also decrease correlatively with the severity of trauma [73–75]. Of these, factor V activity decreases more than the other factor activities [73–75]. Together with decreased fibrinogen levels, decreased factor V levels were detected in patients at accident sites [74]. Platelet counts are seldom reduced to a critical level (<100 × 10^9^/L) in patients on arrival at emergency departments and decrease slower than do fibrinogen levels [1].

## Conclusions

The pathophysiology of trauma-induced coagulopathy consists of coagulation activation, hyperfibrino(geno)lysis, and consumption coagulopathy. These pathophysiological mechanisms are characteristic to DIC with the fibrinolytic phenotype.

## Abbreviations

DIC: Disseminated intravascular coagulation
PAI: Plasminogen activator inhibitor
t-PA: Tissue-plasminogen activator
HMGB-1: High mobility group box nuclear protein 1, TM, thrombomodulin
FDP: Fibrin/fibrinogen degradation products.
